# Enhanced Performance of In_2_O_3_/SnO_2_ Dual-Channel Thin-Film Transistor by Heterostructure Strategy

**DOI:** 10.3390/ma19204284

**Published:** 2026-10-11

**Authors:** Pengbo Guo, Wenwu Pan, Kaijie Gu, Ziyue Hu, Shihao Wei, Yueyan Tian, Hailin Jing, Xiaochao Wu, Ying Li, Wengao Pan

**Affiliations:** 1 Henan Key Laboratory of Advanced Conductor Materials, Institute of Materials, Henan Academy of Sciences, Zhengzhou 450046, China; 2 School of Materials Science and Engineering, Zhengzhou University, Zhengzhou 450001, China; 3 Materials Department, Crane Semicon Technology Co., Ltd., Shanghai 201304, China; 4 Department of Mechanical and Electrical Engineering, Shenzhen Polytechnic University, Shenzhen 518055, China

**Keywords:** In_2_O_3_/SnO_2_, dual-channel, heterojunction, thin-film transistor

## Abstract

Metal-oxide thin-film transistors (TFTs) are promising for next-generation display backplanes and large-area electronics, while single-channel oxide TFTs often suffer from trade-offs among mobility, leakage current, and switching behavior. Here, an In_2_O_3_/SnO_2_ dual-channel TFT was fabricated by magnetron sputtering to combine the high electron transport capability of In_2_O_3_ with the leakage-suppression characteristics of SnO_2_. Compared with single-layer In_2_O_3_, single-layer SnO_2_, and reverse-stacked SnO_2_/In_2_O_3_ devices, the optimized In_2_O_3_/SnO_2_ TFT exhibits improved overall performance, with a linear mobility of 19.15 cm^2^/Vs, a subthreshold swing of 0.86 V/dec, an on/off current ratio of 3.58 × 10^6^ and an optimized threshold voltage. The mobility is approximately 2.2 times higher than that of the single-layer In_2_O_3_ TFT, accompanied by obviously enhanced positive and negative stress stabilities. Furthermore, structural, chemical and optical analyses indicate that the enhanced performance can be attributed to the smooth heterointerface, favorable band alignment, and interfacial carrier redistribution between In_2_O_3_ and SnO_2_. The superior performance highlights the potential of the In_2_O_3_/SnO_2_ bilayer architecture for high-performance display-backplane switching and other large-area oxide-electronics applications.

## 1. Introduction

Thin-film transistors (TFTs) enable wearable active-matrix sensor arrays [1] and are widely investigated for display backplanes and integrated circuits [2]. Metal-oxide semiconductors have attracted considerable interest as TFT channel materials because of their optical transparency and tunable electrical properties. However, achieving high mobility while maintaining excellent stability and a suitable threshold voltage remains challenging [3]. Channel designs that balance trade-off relations among mobility, threshold voltage, and operational stability are therefore highly desirable.

Strategies to address this challenge include elemental doping, process optimization, and multilayer channel engineering. Among them, heterojunction engineering provides additional scope to regulate carrier transport, defect states, and switching behavior through interfacial band alignment and carrier redistribution [4]. For example, the mobility of In_2_O_3_ TFT is enhanced from 59.9 cm^2^/Vs to 81.5 cm^2^/Vs by incorporating cation-doped ZnSnO layers, accompanied by optimized subthreshold swing and no significant improvement in stress stabilities [5]. Lee et al. investigated the thickness-dependent electrical characteristics of ZnO/SnO_2_ heterostructure TFTs and achieved a high *I*_on_/*I*_off_ ratio of approximately 10^10^ by optimizing the multilayer channel architecture by PLD process [6]. In addition, Yu et al. demonstrated a synergistic optimization of threshold voltage and current switching characteristics in In_2_O_3_/IGO bilayer-channel TFTs by combining the carrier transport capability of In_2_O_3_ with the effective carrier-control characteristics of IGO. Although the *I*_on_/*I*_off_ ratio was enhanced by approximately four orders of magnitude compared with the single-layer In_2_O_3_ device, this improvement was accompanied by a reduction in field-effect mobility from 3.2 cm^2^/Vs to 2.6 cm^2^/Vs [7]. Some studies have examined how band alignment and interfacial carrier distribution affect transport in oxide heterojunction TFTs, the mechanism for balancing device mobility and stability in heterostructure engineering remains to be explored [8,9,10]. Overall, the effectiveness of heterojunction channel engineering depends not only on the heterostructure configuration but also on the electronic properties of the constituent oxides and their interfacial compatibility.

The electronic structure and carrier transport characteristics of oxide semiconductors provide a basis for channel-material design [11]. Their electronic properties, including the electronic structures and band gaps of In_2_O_3_ [12,13] and SnO_2_ [14,15], have also been investigated, providing a basis for considering their band alignment when combined in a heterostructure. These material-level findings motivate an investigation of band alignment and possible charge redistribution when the two oxides are combined [16]. In addition, the stacking sequence determines the gate-side channel and the coupling between interfacial carrier redistribution and the gate field, and then strongly influencing threshold voltage, carrier transport, and switching behavior. Comparative investigation of the stacking sequences is therefore necessary for understanding how the channel configuration affects device performance.

In this work, we report a sputtered In_2_O_3_/SnO_2_ dual-channel TFT that synergistically combines the high mobility of In_2_O_3_ with the stability advantages of SnO_2_. The optimized device exhibits a field-effect mobility of 19.15 cm^2^/Vs, more than twice that of the counterpart devices, and excellent bias stability. Combined structural and electrical analyses further clarify the band alignment and interfacial charge-transport mechanism, demonstrating that the In_2_O_3_/SnO_2_ heterostructure is an effective strategy for achieving high mobility while maintaining favorable switching characteristics and stabilities in oxide TFTs.

## 2. Experiment Details

**Figure 1:**
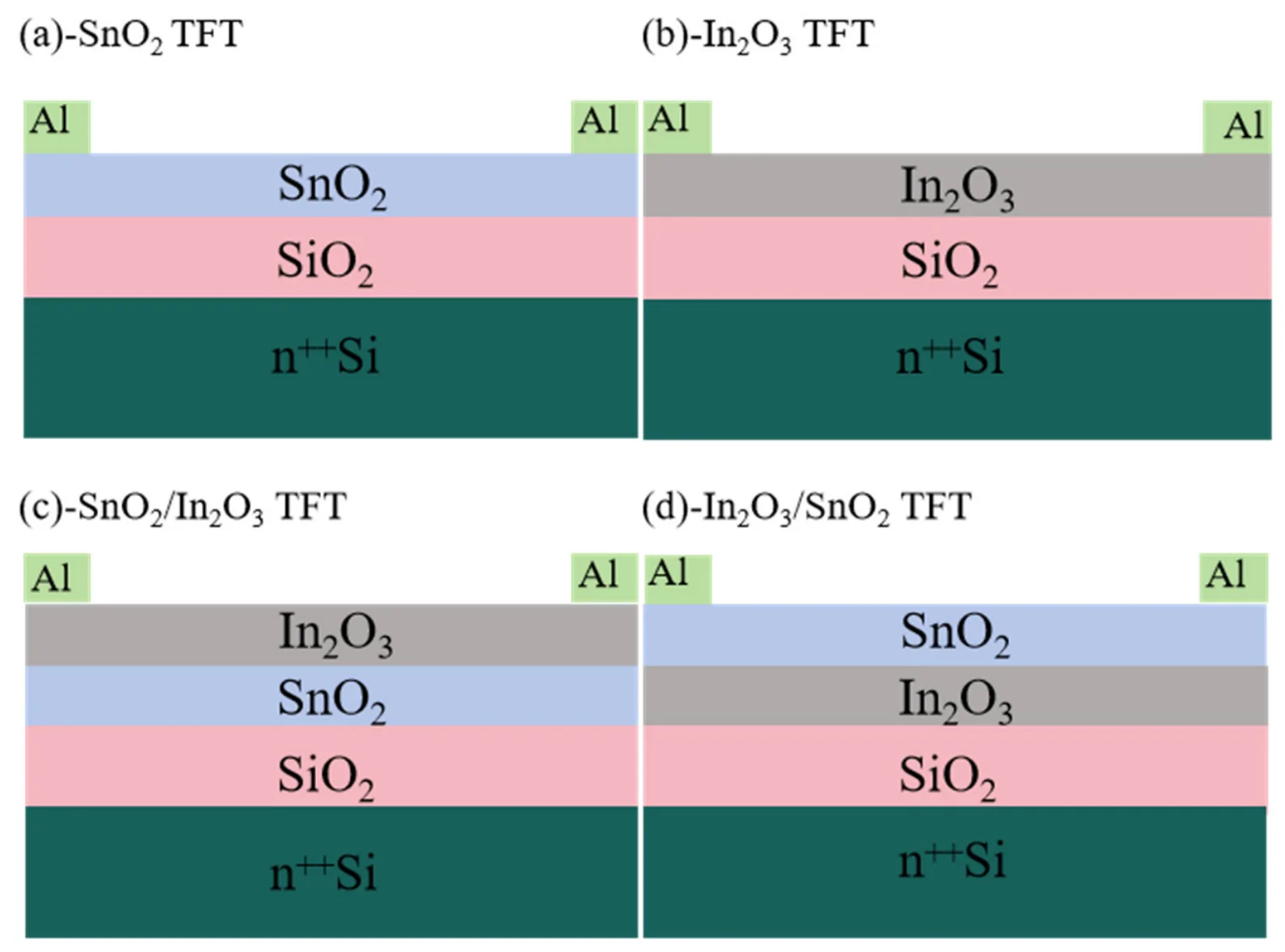
Device structures of (**a**) SnO_2_ TFT, (**b**) In_2_O_3_ TFT, (**c**) SnO_2_/In_2_O_3_ TFT, and (**d**) In_2_O_3_/SnO_2_ TFT.

In_2_O_3_ and SnO_2_ films were fabricated on a glass substrate by a magnetron sputtering technique using corresponding high-purity ceramic targets. Before deposition, glass substrates were sequentially cleaned in acetone, ethanol and deionized water via ultrasonic treatment to remove surface contaminants. DC sputtering power, background and sputtering pressure were set as 100 W, 5 × 10^−4^ Pa and 0.3 Pa, respectively. Sputtering was performed in an Ar/O_2_ gas mixture of 20 sccm/30 sccm, subsequently, the films were annealed at 300 °C in air ambience for 60 min to achieve film densification. The TFTs were fabricated on Si/SiO_2_ substrates using a bottom-gate top-contact structure with different channel configurations, as shown in Figure 1**,** where n^++^ Si serves as the gate electrode and 100 nm thermally oxidized SiO_2_ acts as the gate insulator. The single-layer In_2_O_3_ and SnO_2_ films used for the TFT channel have thicknesses of 5 nm and 15 nm, respectively. The sputtering parameters and annealing process for the channels were consistent with those employed for film characterization. The Al source and drain electrode layers were deposited by thermal evaporation coating technique. All the channels and top electrodes were patterned by a shadow mask with W/L = 500 μm/100 μm.

For material-level characterization, 50 nm In_2_O_3_ and SnO_2_ reference films were prepared to ensure sufficient signal intensity. These thicker films were used only to obtain reference information on chemical states, optical bandgap, work function, and approximate energy-level parameters, and were not assumed to fully represent the thickness-dependent structural or morphological properties of the 5 nm In_2_O_3_ and 15 nm SnO_2_ channel layers used in the TFTs. The crystallographic information and microstructures of In_2_O_3_ and SnO_2_ films were characterized by X-ray diffraction (XRD, Malvern Panalytical, Malvern, UK) and field-emission scanning electron microscope (FE-SEM, Helios 5 CX, Waltham, MA, USA), respectively. The lattice structure and crystal state were checked using high-resolution transmission electron microscopy (HRTEM, FEI-Talos F200S, Waltham, MA, USA). Atomic force microscopy (AFM, Bruker Dimension Icon, Santa Barbara, CA, USA) was used to investigate the surface morphology and roughness of the films. The chemical state, work function and secondary electron cutoff edge of the films were detected by X-ray/ultraviolet photoelectron spectroscopy (XPS/UPS, Thermo Scientific K-Alpha, Waltham, MA, USA), and a He I light source with energy of 21.22 eV was used for UPS measurement. Optical transmittance spectra and bandgap properties were studied using an ultraviolet–visible spectrophotometer (Agilent, Cary5000, Santa Clara, CA, USA). TFT performances were measured with a dual-channel digital source meter (Keithley 2636B, Solon, OH, USA) combined with a probe station (CINDBEST, CS4, Shenzhen, China) at room temperature.

## 3. Results and Discussion

**Figure 2:**
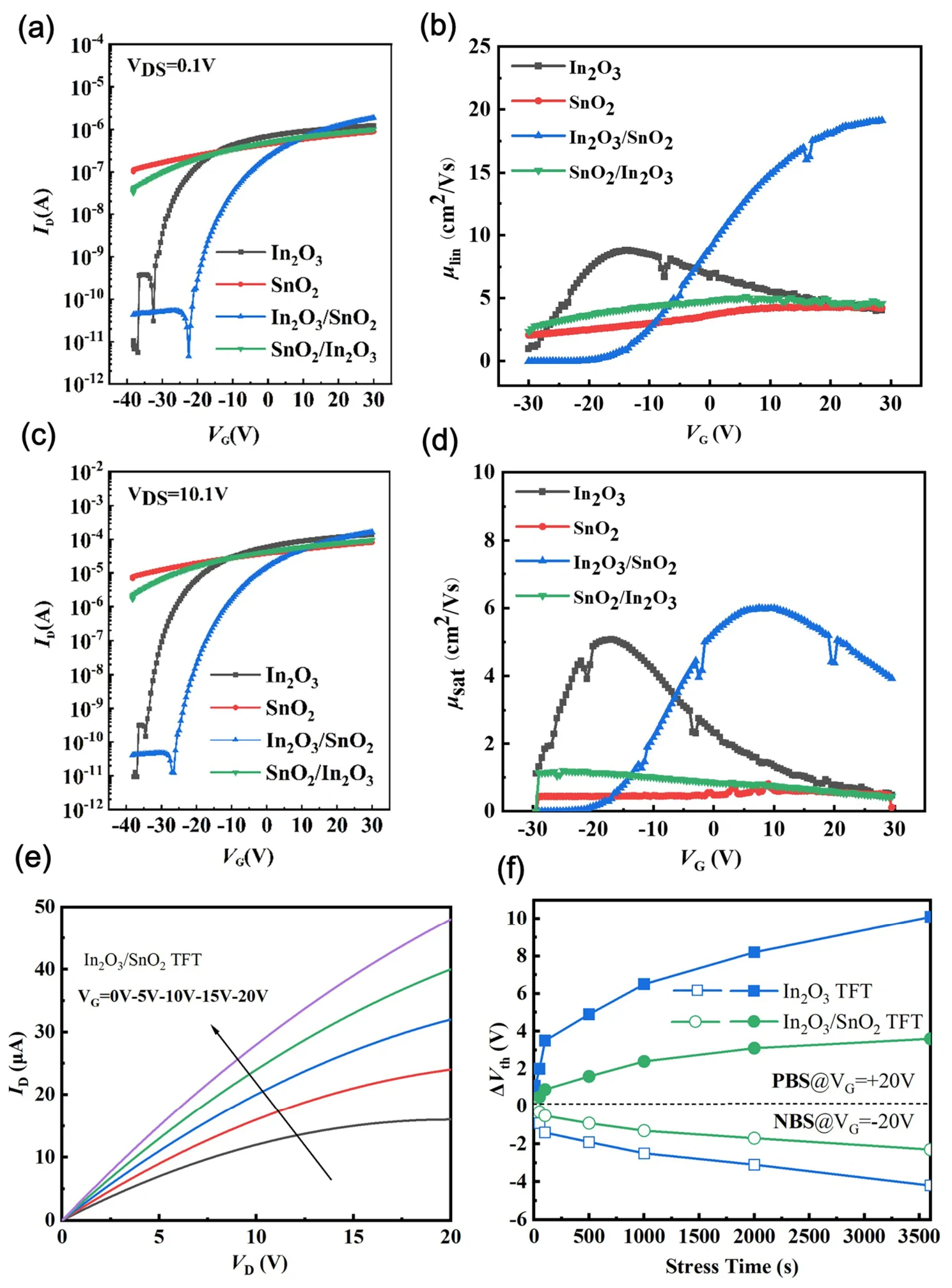
The TFT transfer characteristic curves of single-layer In_2_O_3_, SnO_2_, and dual-layer In_2_O_3_/SnO_2_, and SnO_2_/In_2_O_3_ channels at (**a**) *V*_D_ = 0.1 V and (**c**) *V*_D_ = 10.1 V, along with the corresponding (**b**) linear mobility and (**d**) saturation mobility. (**e**) The output characteristic curves of In_2_O_3_/SnO_2_ TFT. (**f**) The PBS and NBS stabilities of In_2_O_3_ TFT and In_2_O_3_/SnO_2_ TFT.

Figure 2a,b shows the transfer characteristic curves and extracted linear mobility curves of single-layer and heterojunction TFTs investigated in this work at a drain-source voltage (*V*_D_) of 0.1 V. Both single-layer SnO_2_ and SnO_2_/In_2_O_3_ TFTs exhibit no switching characteristics in the current measurement range due to the significantly negative *V*th; In_2_O_3_ TFT possesses a negative *V*th and insufficient on-state current although it can be normally switched. By comparison, a remarkably enhanced on-state current and an optimized *V*th were achieved in dual-channel In_2_O_3_/SnO_2_ TFT, with In_2_O_3_ as the front channel layer and SnO_2_ as the back-channel layer. Figure 2c,d show the transfer characteristic curves and the corresponding saturation mobility curves of the four TFTs at *V*_D_ = 10.1 V. Similar to the curves of *V*_D_ = 0.1 V, the In_2_O_3_/SnO_2_ heterojunction TFT exhibits the most optimal comprehensive performance with the highest saturation mobility. These results indicate the switching characteristics of dual-channel devices strongly depend on the layer stacking sequence, and the relevant mechanisms will be discussed in detail below in conjunction with band structures.

Figure 2e shows the output characteristics of the In_2_O_3_/SnO_2_ TFT, and the absence of pronounced current crowding suggests that severe contact-limited injection is unlikely. However, the contribution from contact effects cannot be completely excluded due to the absent quantitative extraction of contact resistance. In addition, no clear current plateau is observed as *V*_D_ increases, providing no direct evidence for dominant high-field velocity saturation. It has been reported that increasing the positive gate voltage can progressively fill interfacial or defect-related trap states, thereby reducing transport barriers and improve the device mobility [17]. Accordingly, the observed discrepancy between the linear and saturation mobilities may arise from the combined effects of gate-bias-dependent trap-state dynamics and contact resistance. Figure 2f summarizes the positive bias stress (PBS) and negative bias stress (NBS) stabilities of In_2_O_3_ TFT and In_2_O_3_/SnO_2_ TFT under ±20 V gate voltage, respectively. The In_2_O_3_/SnO_2_ TFT exhibits substantially smaller threshold-voltage shift (Δ*V*_th_) under both stress conditions. After 3600 s, the PBS and NBS induced Δ*V*_th_ were approximately +3.6 V and −2.3 V, respectively, for the In_2_O_3/_SnO_2_ TFT, compared with +10.1 V and −4.2 V for the In_2_O_3_ TFT. These results indicate that the SnO_2_ back-channel layer contributes to improved electrical stability under both PBS and NBS. The improvement may be associated with reduced bias-induced charge trapping and suppressed interaction between the back-channel surface and ambient species [18].

For further comparison, some key electrical parameters including threshold voltage, subthreshold swing (*SS*), switching current ratio, and mobility were calculated according to the following equations [19,20].

(1) $$\begin{array}{c} I_{D}=\frac{W}{L}\mu_{\mathrm{lin}}C_{\mathrm{ox}}\left[\left(V_{G}-V_{\mathrm{th}}\right)V_{D}-\frac{V_{D}^{2}}{2}\right] \end{array}$$

(2) $$I_{D}=\frac{W}{2L}\mu_{\mathrm{sat}}C_{\mathrm{ox}}{\left(V_{G}-V_{\mathrm{th}}\right)}^{2}$$

(3) $$\mathrm{SS}=\frac{dV_{G}}{d(\log I_{D})}$$

where *W* and *L* represent the width and length of the device, respectively; *I*_D_, *V*_D_, *V*_G_, and *C*_ox_ denote the current between the TFT source and drain, the source-drain voltage, and the gate-source voltage and gate capacitance per unit area, respectively. The *C*_ox_ (34.5 nF/cm^2^) is calculated solely from the 100 nm SiO_2_ gate insulator. The *V*th is confirmed by constant current method, which is equal to the *V*_G_ corresponding to an *L*/*W*-normalized *I*_D_ of 1 nA for *V*_D_ = 0.1 V and 100 nA for *V*_D_ = 10.1 V, respectively. The current ratio refers to the ratio of the on-state current to the off-state current (*I*_on_/*I*_off_).

Table 1 summarizes the specific electrical parameters of the four TFTs. The In_2_O_3_/SnO_2_ TFT exhibits an optimal comprehensive performance, with *μ*_sat_ of 5.98 cm^2^/Vs, *μ*_lin_ of 19.15 cm^2^/Vs, *SS* of 0.86 V/dec, *V*th of −17.5 V and *I*_on_/*I*_off_ of 3.58 × 10^6^. In particular, the linear mobility was enhanced at least two times compared with the other three counterpart devices. It should be noted that contact resistance was not quantitatively extracted, and the derived linear mobility may therefore be influenced by both gate-bias-dependent trap-state dynamics and contact effects. The enhancement in comprehensive performance can be plausibly attributed to the joint actions of complementary material properties and matching band structures of In_2_O_3_ and SnO_2_. On one hand, a heterojunction quantum well may form at their interface, effectively reducing scattering and facilitating electron transport [21]. On the other hand, the In_2_O_3_ layer in the front channel and SnO_2_ layer in the back channel serve as the high mobility layer and interface stability layer respectively [22,23]. The strongly negative *V*th may arise from the high electron concentration, oxygen-vacancy-related donor states, and interfacial carrier redistribution in the In_2_O_3_/SnO_2_ heterostructure. This depletion-mode-like operation may increase standby power consumption and complicate circuit integration. The negative *V*th is an important limitation of the present heterostructure TFT although it shifts *V*th from −30.0 V for the single-layer In_2_O_3_ TFT to −17.5 V, and oxygen partial-pressure control, dielectric/channel interface treatment, and back-channel passivation are possible strategies for future optimization.

**Figure 3:**
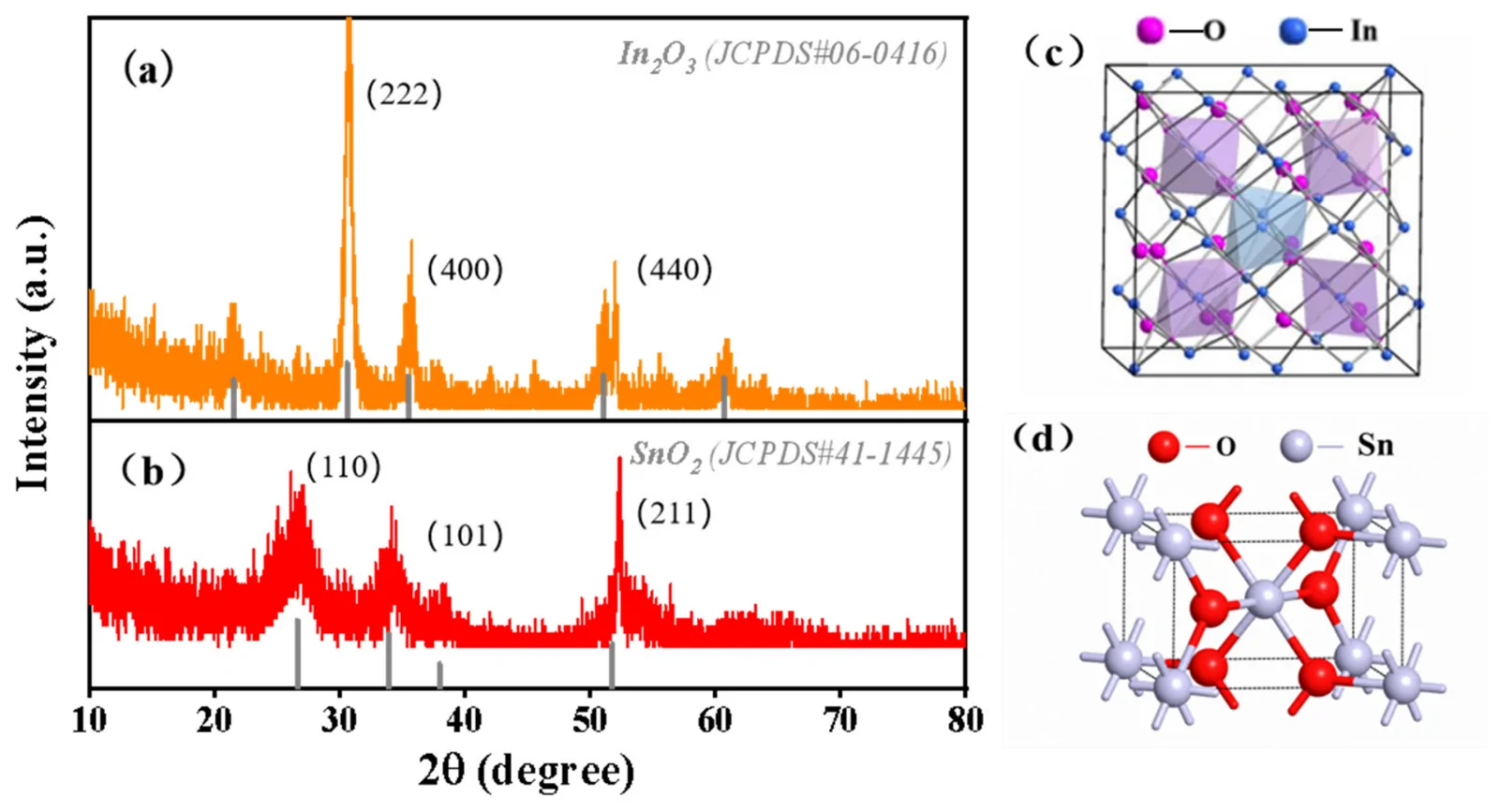
(**a**,**b**) GIXRD spectra of SnO_2_ and In_2_O_3_ thin films and (**c**,**d**) their corresponding crystal structures.

To investigate the mechanism underlying the performance enhancement of In_2_O_3_/SnO_2_ heterojunction TFTs, several material characterization techniques were employed. Firstly, GIXRD spectra of the In_2_O_3_ and SnO_2_ films were measured within the 2θ range of 10–80°. Both films exhibit insufficient crystallinity or even nanocrystalline characteristics, and no detectable impurity phases were identified, as shown in Figure 3a,b. In addition, In_2_O_3_ film shows three major diffraction peaks, at 2θ = 30.6°, 35.5°, and 50.9°, corresponding to the (222), (400), and (440) crystal planes of the cubic bixbyite In_2_O_3_ phase (#06-0416), respectively, as shown in Figure 3a,c. Similarly, SnO_2_ film also shows three major diffraction peaks at 2θ = 26.5°, 33.8°, and 51.7°, corresponding to the (110), (101), and (211) crystal planes of the tetragonal rutile-phase SnO_2_ (JCPDS #41-1445) [24], as shown in Figure 3b,d. To further compare the differences, the grain size can be quantitatively calculated using the Scherrer Equation [25,26].

(4) $$D=\frac{0.9\lambda }{\beta \cos\theta }$$

Here, *D*, *λ*, *θ*, and *β* represent the grain size, incident light wavelength, diffraction angle, and half-width at half maximum (FWHM) of the diffraction peak, respectively. Based on the structural analysis, the average grain sizes of the SnO_2_ and In_2_O_3_ films are estimated to be approximately 10 and 18 nm, respectively, for the 50 nm reference films.

**Figure 4:**
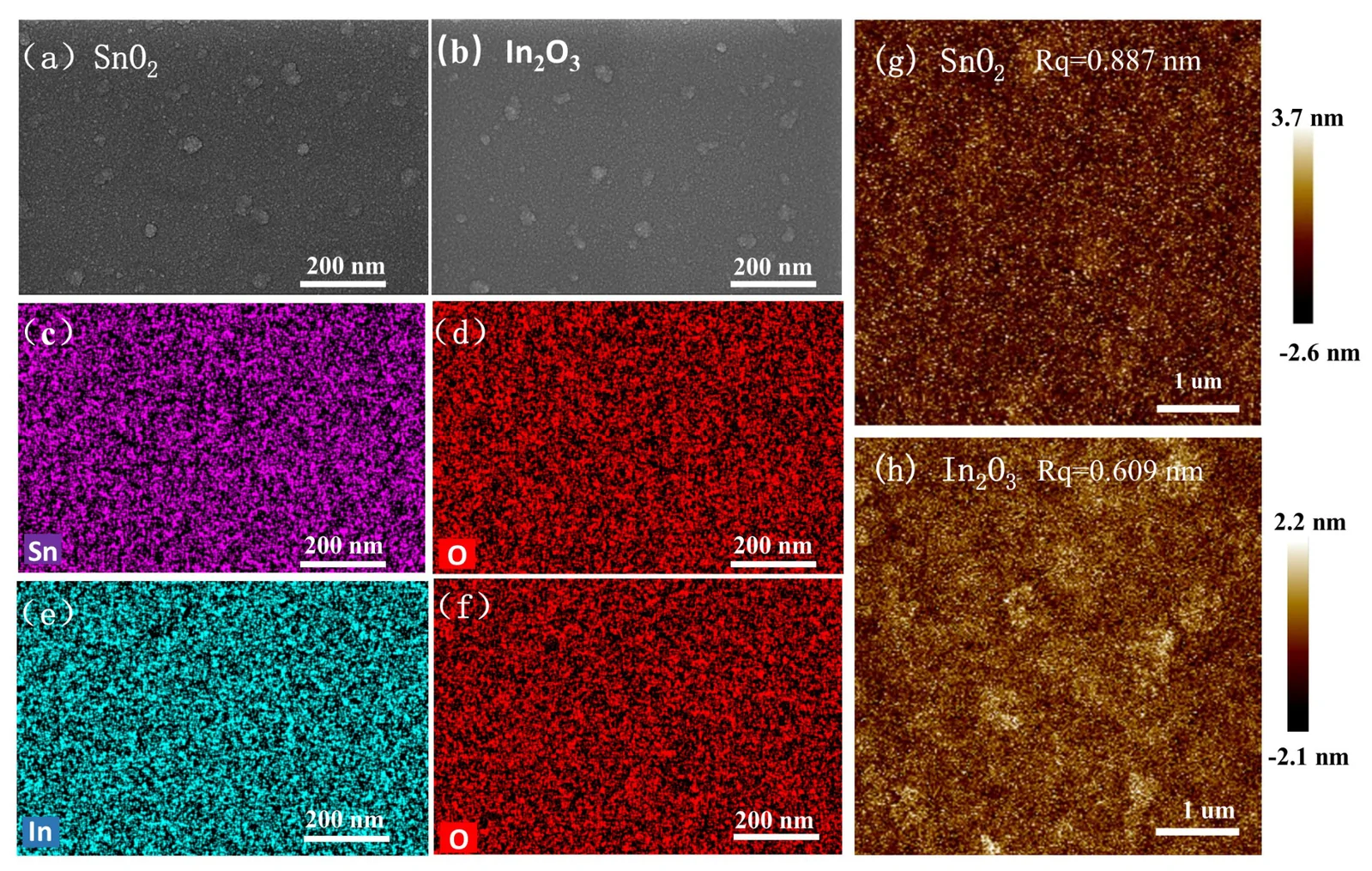
(**a**,**b**) The SEM, (**c**–**f**) EDS, and (**g**,**h**) AFM results of the SnO_2_ and In_2_O_3_ films.

Figure 4 shows SEM, EDS, and AFM images of the SnO_2_ and In_2_O_3_ films. The SEM results reveal some particle aggregates on the surface of the two films with indistinguishable grain boundaries, exhibiting weak crystallization characteristics. The grain sizes of them can be roughly estimated to be 10~20 nm (Figure 4a,b), and the specific differences cannot be further distinguished due to the limited amplification capability of SEM and/or the near resolution limit of grain size, consistent with the GIXRD result. The EDS results in Figure 4c–f indicate that both the SnO_2_ and In_2_O_3_ films exhibit a uniform elemental distribution with no localized element depletion or enrichment. The measured root-mean-square roughness (*R_q_*) values for the SnO_2_ and In_2_O_3_ films are 0.89 nm and 0.61 nm, respectively, suggesting both films exhibit highly smooth surfaces, evident from the AFM results in Figure 4g,h. The smooth surfaces are favorable for achieving not only an excellent ohmic contact between electrodes and active layers, but also a high quality of interface around electron transport channels, and then beneficial to utilizing the positive effects of the heterojunction interface to enhance the comprehensive performance of TFT. It is worth noting that the XRD, SEM, and AFM results obtained from the 50 nm reference films are used to describe the general structural and surface characteristics of the deposited materials. Because crystallinity, grain size, and surface morphology can depend strongly on film thickness, these results are not directly extrapolated to the ultrathin 5 nm/15 nm TFT channel layers.

**Figure 5:**
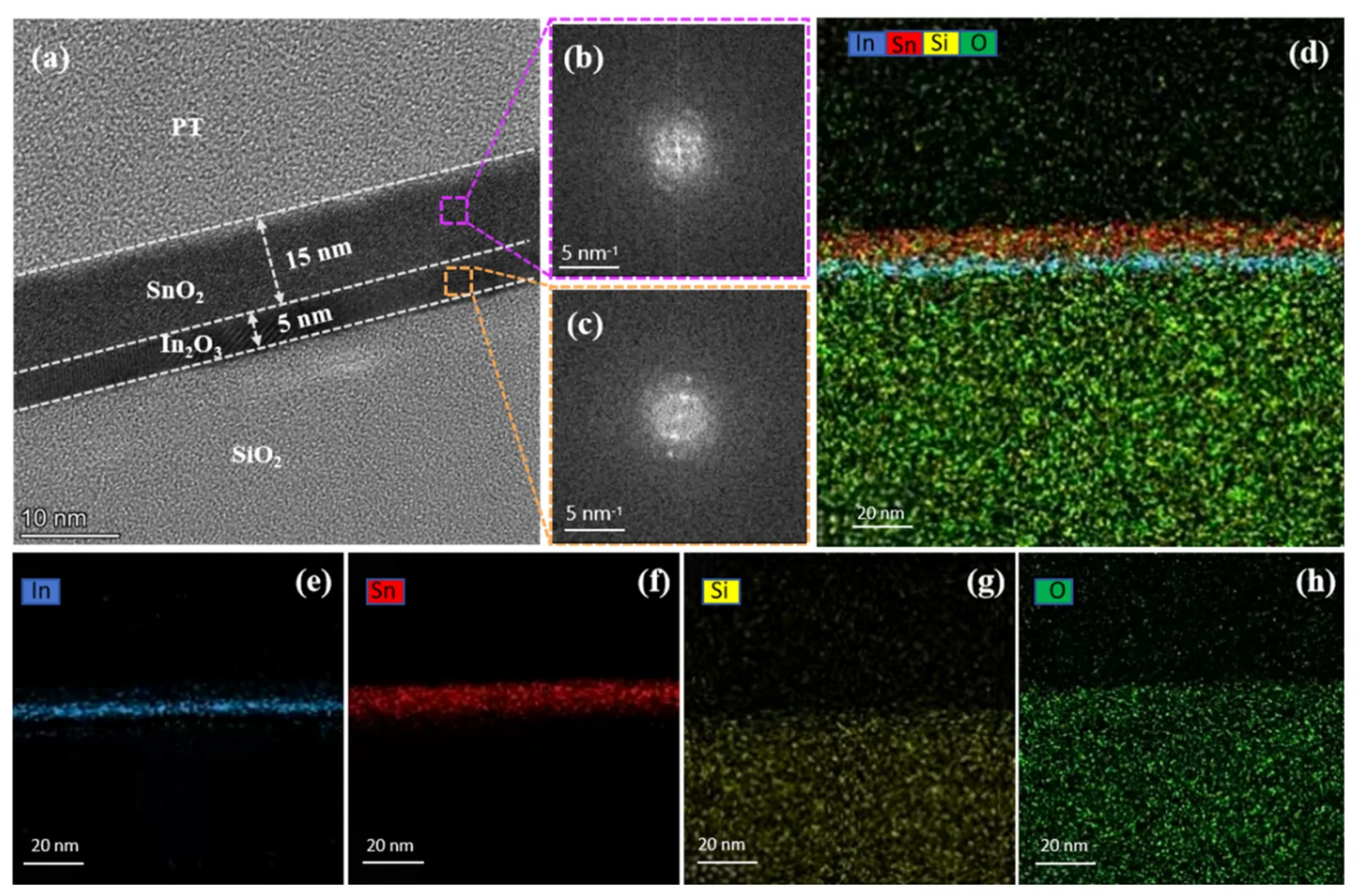
(**a**) HRTEM images of the dual-active-layer thin films, corresponding FFT spectra (**b**,**c**) and EDS energy spectra (**d**–**h**).

In contrast to the 50 nm reference films, the cross-sectional HRTEM, fast Fourier transform (FFT), and energy-dispersive X-ray spectroscopy (EDS) analyses shown in Figure 5 were performed on the device-relevant 5 nm In_2_O_3_/15 nm SnO_2_ bilayer structure and provide the direct structural evidence for the actual TFT channel. Figure 5a demonstrates clear, continuous morphological features at both the SiO_2_/In_2_O_3_ and In_2_O_3_/SnO_2_ interfaces, with no significant diffusion observed between the layers. The thicknesses of SnO_2_ and In_2_O_3_ are confirmed to be approximately 15 nm and 5 nm, respectively. The smooth, non-diffusive and continuous interfaces reduce interface defect state density and enhance carrier transport between different channels, providing an optimal structural foundation for the formation of two-dimensional electron gas (2DEG). FFT analyses in Figure 5b,c further verify the very weak crystal structures in both In_2_O_3_ and SnO_2_ films. Figure 5d–h show the EDS mapping results of the In_2_O_3_/SnO_2_ layered film. The elemental distribution profiles exhibit clear layer-dependent characteristics, with Si, Sn, and In predominantly localized in the substrate, SnO_2_, and In_2_O_3_ layers, respectively. The sharp interfacial transitions and absence of significant diffusion zones are consistent with TEM observations, confirming the successful fabrication of a well-defined and compositionally uniform In_2_O_3_/SnO_2_ dual-active-layer thin film by magnetron sputtering and in situ deposition.

**Figure 6:**
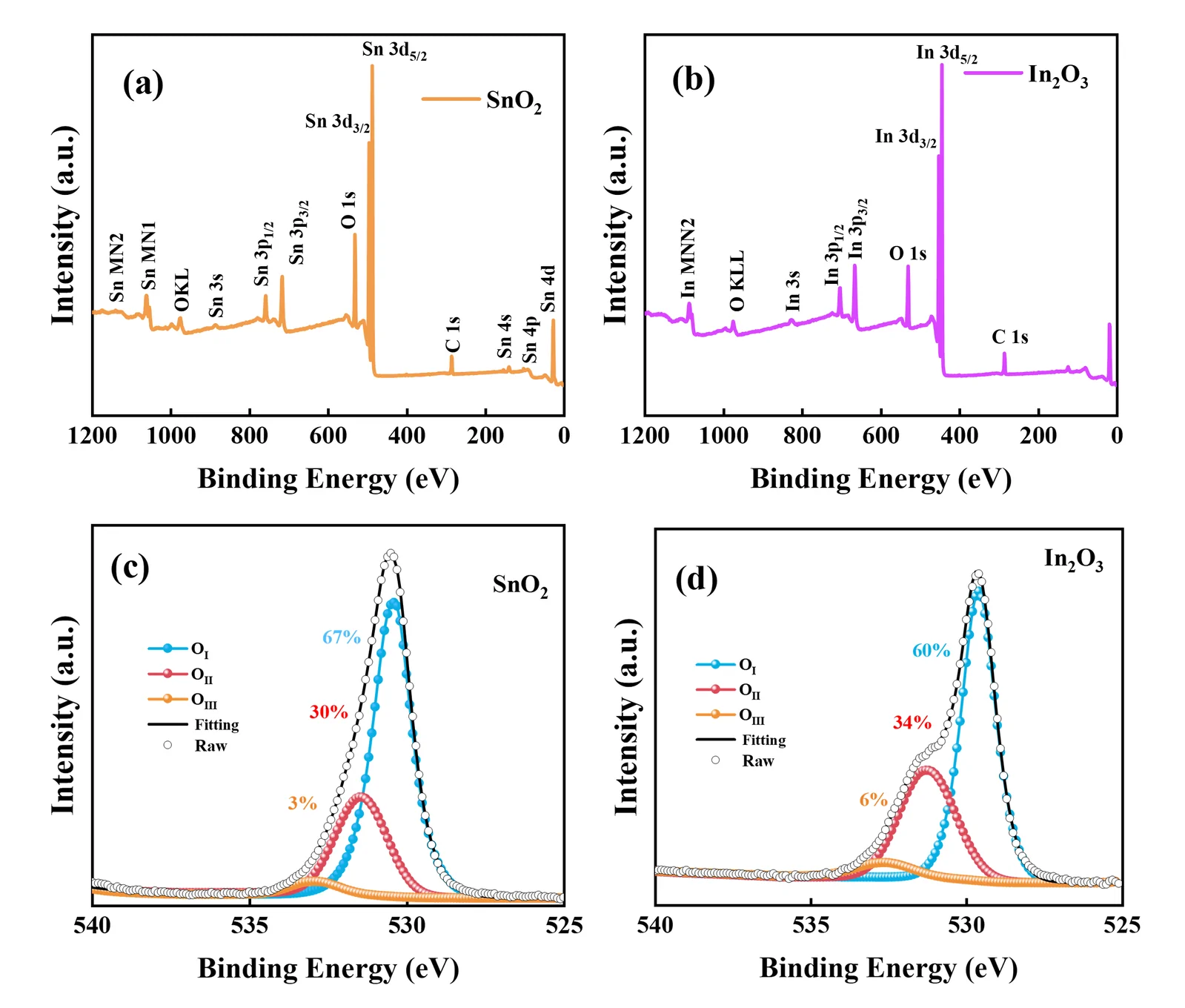
(**a**,**b**) Full XPS spectra and (**c**,**d**) O 1s peak fitting plots of SnO_2_ and In_2_O_3_ thin films.

Figure 6 presents the XPS analysis results and O 1s peak fitting plots for the SnO_2_ and In_2_O_3_ thin films. Figure 6a,b show the full XPS spectra of the SnO_2_ and In_2_O_3_ films, respectively, and they clearly reveal the binding energy peaks corresponding to elements such as In, Sn, and O. Apart from the inevitably present surface-adsorbed carbon, no other impurity elements were detected. Figure 6c,d display the O 1s energy spectra and the corresponding deconvolution results of the two films. The O 1s spectra were decomposed into three characteristic peaks employing the Gaussian–Lorentz fitting method [27], i.e., lattice oxygen (O_I_), oxygen vacancy (O_II_), and hydroxyl-adsorbed oxygen (O_III_). For SnO_2_, the binding energies of the fitted peaks are 530.4 eV (O_I_), 531.4 eV (O_II_), and 532.9 eV (O_III_) [28], and they are 529.6 eV (O_I_), 531.2 eV (O_II_), and 532.6 eV (O_III_) for In_2_O_3_ [29]. According to the integral area, the relative contents of oxygen vacancy in SnO_2_ and In_2_O_3_ films are 30% and 34%, respectively. It indicates oxygen vacancies are more easily formed in In_2_O_3_ film, plausibly due to the weak bond connection between In and O. Oxygen-vacancy-related states may facilitate charge transport and enhance field-effect mobility under specific conditions [30]. By comparison, Sn-O bond is stronger and more favorable for the enhancement of device stability, when it serves as the back-channel layer.

**Figure 7:**
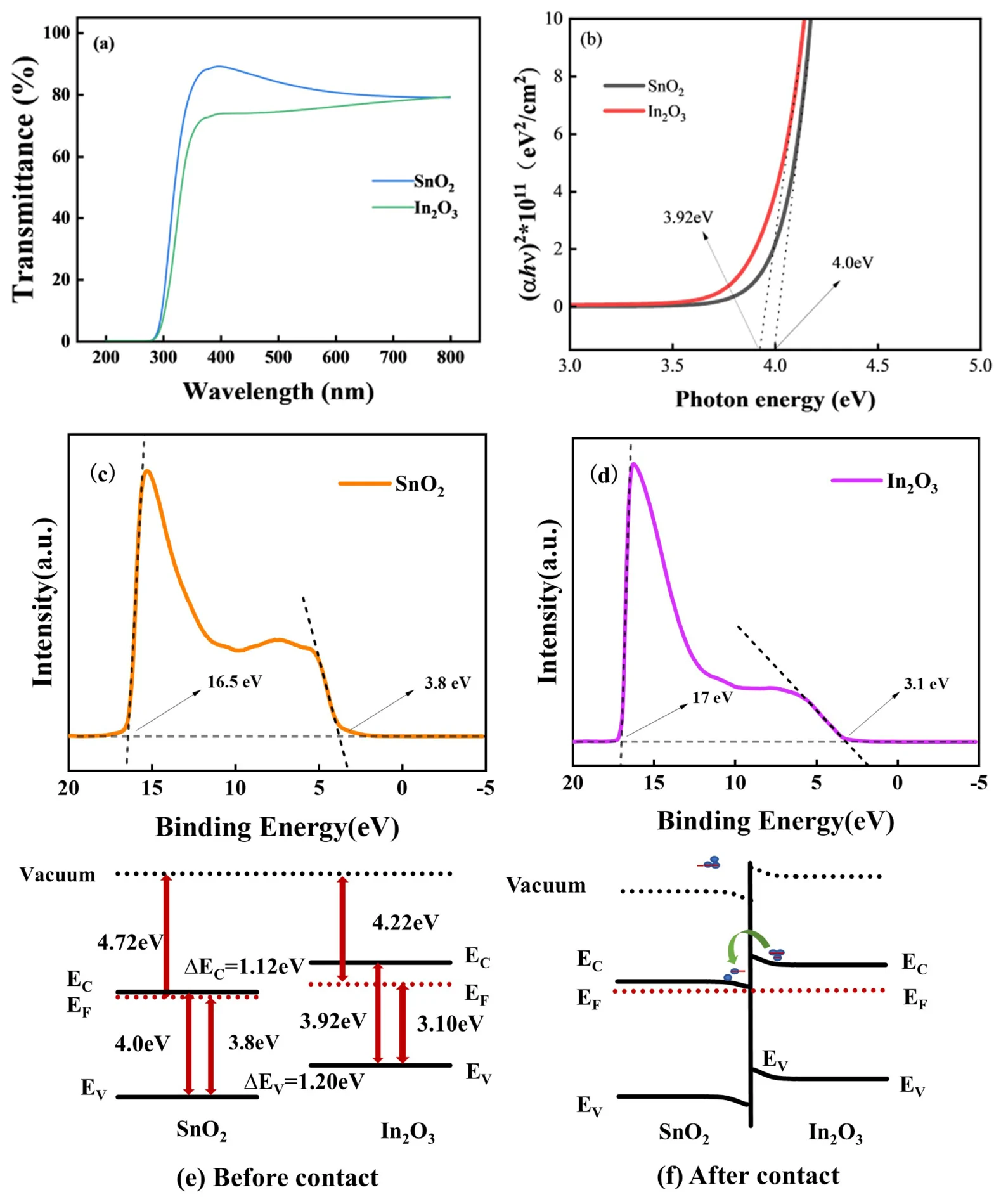
(**a**) The optical transmittance spectra, (**b**) Tauc plots, and (**c**,**d**) UPS spectra of the SnO_2_ and In_2_O_3_ thin films, along with (**e**,**f**) the energy band structure diagrams before and after contact between them.

Figure 7a shows the optical transmittance of the 50 nm SnO_2_ and In_2_O_3_ reference films, which were used to derive the optical band gaps and, together with the UPS results, construct an approximate band-alignment model. Possible thickness-dependent shifts in the ultrathin device channels cannot be completely excluded. In the wavelength range of 380–800 nm, optical transmittance of In_2_O_3_ films is nearly 80%, while that of SnO_2_ generally exceeds 80%, indicating a certain application potential in transparent displays. The optical bandgap (*E*_g_) is further derived from the optical transmittance data based on the Tauc plot and the Formula (5) [31,32,33]. As shown in Figure 7b, *E*_g_ is 4.0 eV for SnO_2_ and 3.92 eV for In_2_O_3_. Figure 7c,d present the UPS spectra of the SnO_2_ and In_2_O_3_ films. To separate the secondary electron cutoff edge from the instrument’s cutoff edge and facilitate precise work function analysis, the spectra were corrected using a −5 V bias voltage [34]. The work functions of SnO_2_ and In_2_O_3_ film have been Fermi-calibrated and are further extracted using Formula (6) [35,36].

(5) (*ahv*)^2^ = *A*(*hv* − *E_g_*)

(6) ϕ = hν − E_SECO_

where α, *h*, *v*, *A*, *E_g_*, *ϕ* and *E_SECO_* represent the absorption coefficient, Planck constant, incident light frequency, Tauc constant, bandgap width, work function, and secondary electron cutoff energy, respectively.

In the equilibrium state, the features of heterojunction interface primarily depend on the band structure between the two channels, including differences in the conduction band minimum (*E*_CBM_), valence band maximum (*E*_VBM_), and Fermi energy level (*E*_F_) [37]. This matched band structure induces band bending and forms a potential well or barrier at the interface, and then promotes the electron localization in a certain transport channel [38].

To elucidate the physical mechanisms underlying the performance enhancement of the double-layer In_2_O_3_/SnO_2_ TFT and its carrier transport behavior, the band structures of SnO_2_ and In_2_O_3_ before and after contact were analyzed, as shown in Figure 7e,f. Due to the significant CBM difference (*∆E_C_* = 1.12 eV) between SnO_2_ and In_2_O_3_, a portion of electrons from the In_2_O_3_ film migrate to the SnO_2_ side after contact by Fermi level alignment mechanism, forming a depletion layer at the In_2_O_3_ interface while increasing electron concentration around the SnO_2_ interface. The creation of a potential well at the interface may facilitate a possible energetic basis for 2DEG carrier confinement electron accumulation and enhanced electron concentration and mobility [39]. This heterojunction interface serves as an efficient carrier transport channel, effectively mitigating the effects of interface defects and impurity scattering on charge transport [40]. Additionally, the presence of potential barrier on the side of the SnO_2_ back channel is also beneficial to the positive shift in the threshold voltage [41,42], which aligns well with the performance result. It can be expected that the In_2_O_3_/SnO_2_ heterojunction structure functions as a dual-channel parallel conduction pathway, that is, electrons transport simultaneously at the interfaces of In_2_O_3_ and SnO_2_, as well as between In_2_O_3_ and SiO_2_. From the perspective of multifunctional collaboration, the front layer of In_2_O_3_ serves as a high mobility channel, and the back channel of SnO_2_ plays the role of interface/environment stability layer. The joint actions of heterojunction interface and multifunctional collaboration layer configuration result in a significant enhancement of the device’s comprehensive performance.

**Figure 8:**
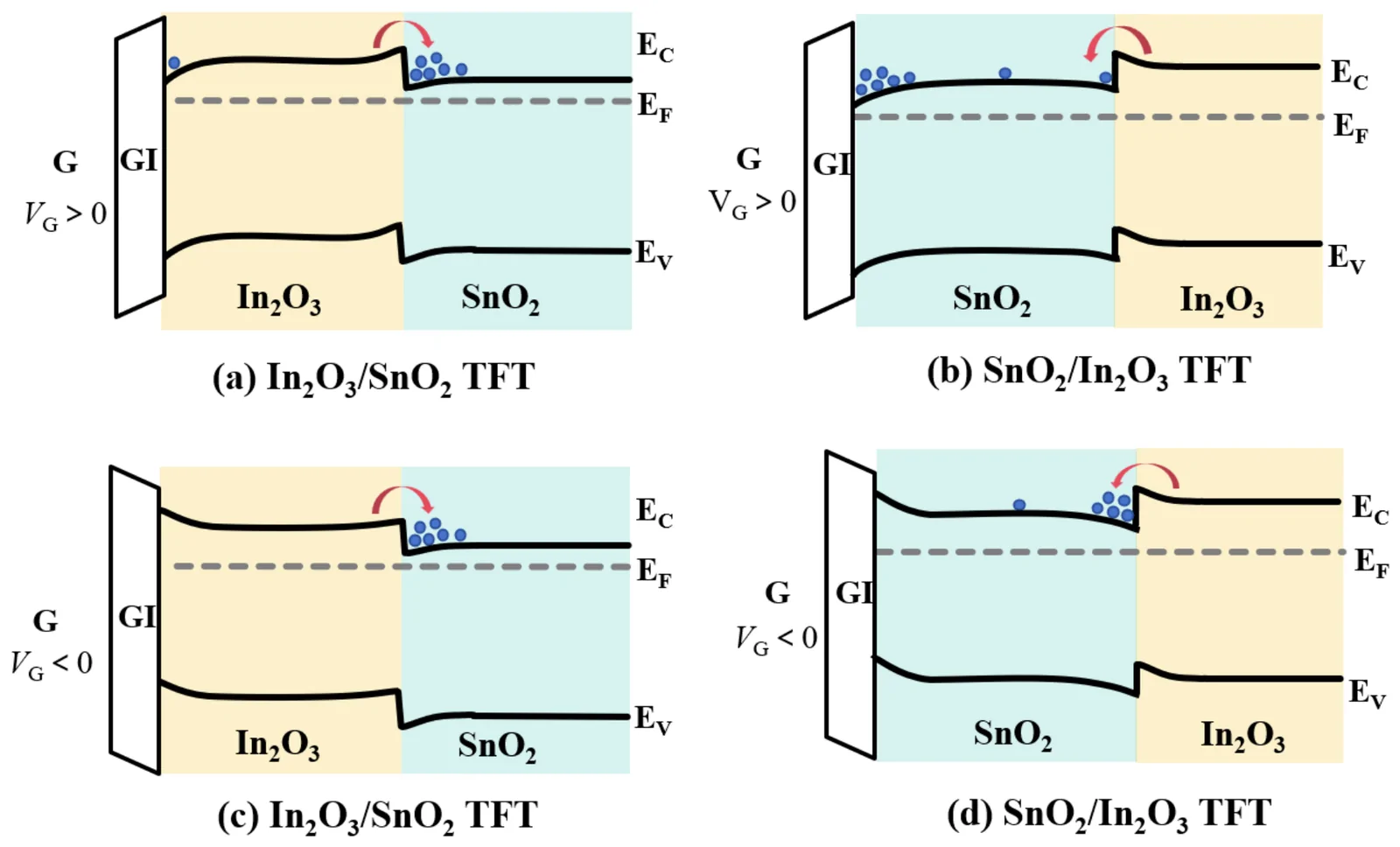
Schematic diagram of band structures of In_2_O_3_/SnO_2_ TFT and SnO_2_/In_2_O_3_ TFT under positive (**a**,**b**) and negative (**c**,**d**) gate biases.

To further clarify the pronounced stacking-order dependence, Figure 8 schematically compares the band bending and carrier distribution of the In_2_O_3_/SnO_2_ and SnO_2_/In_2_O_3_ TFTs under positive and negative gate biases. In bottom-gate dual-channel TFTs, the front channel adjacent to the gate dielectric dominates the direct gate-field modulation, while the upper channel and heterointerface further modify the overall carrier distribution and transport behavior [43,44]. Although reversing the stacking sequence does not change the intrinsic In_2_O_3_/SnO_2_ band alignment, electron transfer from In_2_O_3_ to SnO_2_ is still expected owing to Fermi-level equilibration and the conduction band offset, as indicated by Figure 7f. Under positive *V*_G_, gate-induced accumulation and heterojunction-induced electron enrichment are respectively distributed in In_2_O_3_ and SnO_2_ layers for In_2_O_3_/SnO_2_ TFT. While they are only coexisting within the SnO_2_ channel for SnO_2_/In_2_O_3_ TFT, as shown in Figure 8a,b, under positive *V*_G_ (Figure 8c,d), the interfacial electron transfer lowers the electron density on the In_2_O_3_ side and therefore facilitates depletion of the gate-side channel for the In_2_O_3_/SnO_2_ device, consistent with the positive shift in *V*th from −30.0 V for the single-layer In_2_O_3_ TFT to −17.5 V. In contrast, in the reverse SnO_2_/In_2_O_3_ structure, SnO_2_ becomes the front channel, and the same charge-transfer process enriches the SnO_2_ side of the heterointerface. The electron accumulation in the gate-side channel makes complete depletion of the whole dual-channel stack more difficult, resulting in an extremely negative effective threshold voltage and the absence of clear switching behavior.

## 4. Conclusions

This work addresses the performance limitations of conventional single-layer TFT devices, such as low carrier mobility, unstable threshold voltage, and poor stability. Leveraging the high mobility of In_2_O_3_ and the excellent interface stability of SnO_2_, we develop a method for fabricating In_2_O_3_/SnO_2_ double-channel TFT via DC magnetron sputtering. Superior comprehensive performance is achieved in the In_2_O_3_/SnO_2_ TFT, with high linear mobility of 19.15 cm^2^/Vs, a subthreshold swing (*SS*) of 0.86 V/dec, and an enhanced I_on_/I_off_ compared with the other three counterparts. The significant enhancement of comprehensive performance can be attributed to the joint actions of heterojunction interface and multifunctional collaboration layer configuration. The heterojunction interface may provide a new electron transport channel with low defect scattering. The channel collaboration configuration offers a high mobility layer in the front channel, and enhances the interface/environment stability in the back channel, simultaneously. Although some advances are achieved, the device still exhibits a negative threshold voltage of −17.5 V. Future work will examine oxygen content, dielectric/channel interface treatment, and back-channel passivation to improve threshold-voltage control. Furthermore, scalable patterning, further reliability evaluation, and circuit-level integration should be investigated before the architecture can be considered for display backplanes or other large-area oxide-electronics systems.

## Figures and Tables

**Table 1:** The key electrical parameters of the four TFT devices with different channels.

Channels	*μ*_sat_ (cm^2^/Vs)	*μ*_lin_ (cm^2^/Vs)	*SS* (V/dec)	*V*th (V)	*I*_on_/*I*_off_ (10^5^)
In_2_O_3_	5.03 ± 0.25	8.70 ± 0.45	2.88 ± 0.25	−30.0 ± 1.07	4.4 ± 1.5
SnO_2_	0.80 ± 0.12	4.50 ± 0.20	/	/	/
In_2_O_3_/SnO_2_	5.98 ± 0.33	19.15 ± 1.13	0.86 ± 0.08	−17.5 ± 0.52	35 ± 7.2
SnO_2_/In_2_O_3_	1.20 ± 0.18	4.78 ± 0.23	/	/	/

“/” den otes parameters that could not be extracted due to unsuppressed off-state current within the measured voltage range.

## Data Availability

The original contributions presented in this study are included in the article. Further inquiries can be directed to the corresponding authors.

## References

[1] Baek S., Lee Y., Baek J., Kwon J., Kim S., Lee S., Strunk K.-P., Stehlin S., Melzer C., Park S.-M. (2022). Spatiotemporal measurement of arterial pulse waves enabled by wearable active-matrix pressure sensor arrays. ACS Nano.

[2] Jeon S.P., Jo J.W., Nam D., Kim Y.H., Park S.K. (2025). High-performance metal oxide TFTs for flexible displays: Materials, fabrication, architecture, and applications. Soft Sci..

[3] Yap B.K., Zhang Z., Thien G.S.H., Chan K.Y., Tan C.Y. (2023). Recent advances of In_2_O_3_-based thin-film transistors: A review. Appl. Surf. Sci. Adv..

[4] Wang P., Yang H., Li J., Zhang X., Wang L., Xiao J., Zhao B., Zhang S., Lu L. (2023). Synergistically enhanced performance and reliability of abrupt metal-oxide heterojunction transistor. Adv. Electron. Mater..

[5] Zhu Q., Huang Y., Wu J., Guo M., Ou H., Liu B., Lu X., Chen J., Liang X., Wu Q. (2024). Enhanced performance and stability of atomic layer deposited In_2_O_3_ transistors with multi-function cation-doped ZnSnO layers. IEEE Electron Device Lett..

[6] Lee S.J., Hwang C.S., Pi J.E., Yang J.H., Byun C.W., Chu H.Y., Cho K.I., Cho S.H. (2015). High-performance amorphous multilayered ZnO-SnO_2_ heterostructure thin-film transistors: Fabrication and characteristics. ETRI J..

[7] Yu X., Zhou N., Smith J., Lin H., Stallings K., Yu J., Marks T.J., Facchetti A. (2013). Synergistic approach to high-performance oxide thin film transistors using a bilayer channel architecture. ACS Appl. Mater. Interfaces.

[8] Li X., Ma Z., Li J., Pan W., Liao C., Zhang S., Gao Z., Fu D., Lu L. (2024). Heterojunction-engineered carrier transport in elevated-metal metal-oxide thin-film transistors. J. Semicond..

[9] Huang X., Chen C., Sun F., Chen X., Xu W., Li L. (2024). Enhancing the carrier mobility and bias stability in metal-oxide thin film transistors with bilayer InSnO/a-InGaZnO heterojunction structure. Micromachines.

[10] Jung H.Y., Kang Y., Hwang A.Y., Lee C.K., Han S., Kim D.H., Bae J.U., Shin W.S., Jeong J.K. (2014). Origin of the improved mobility and photo-bias stability in a double-channel metal oxide transistor. Sci. Rep..

[11] Kamiya T., Nomura K., Hosono H. (2010). Present status of amorphous In-Ga-Zn-O thin-film transistors. Sci. Technol. Adv. Mater..

[12] Fuchs F., Bechstedt F. (2008). Indium-oxide polymorphs from first principles: Quasiparticle electronic states. Phys. Rev. B.

[13] King P.D.C., Veal T.D., Fuchs F., Wang C.Y., Payne D.J., Bourlange A., Zhang H., Bell G.R., Cimalla V., Ambacher O. (2009). Band gap, electronic structure, and surface electron accumulation of cubic and rhombohedral In_2_O_3_. Phys. Rev. B.

[14] Presley R.E., Munsee C.L., Park C.-H., Hong D., Wager J.F., Keszler D.A. (2004). Tin oxide transparent thin-film transistors. J. Phys. D Appl. Phys..

[15] Cai X., Zhang P., Wei S.H. (2019). Revisit of the band gaps of rutile SnO_2_ and TiO_2_: A first-principles study. J. Semicond..

[16] Lin Y.H., Faber H., Labram J.G., Stratakis E., Sygellou L., Kymakis E., Hastas N.A., Li R., Zhao K., Amassian A. (2015). High electron mobility thin-film transistors based on solution-processed semiconducting metal oxide heterojunctions and quasi-superlattices. Adv. Sci..

[17] Takagi A., Nomura K., Ohta H., Yanagi H., Kamiya T., Hirano M., Hosono H. (2005). Carrier transport and electronic structure in amorphous oxide semiconductor, a-InGaZnO_4_. Thin Solid Films.

[18] Adl A.H., Kar P., Farsinezhad S., Sharma H., Shankar K. (2015). Effect of sol stabilizer on the structure and electronic properties of solution-processed ZnO thin films. RSC Adv..

[19] Lee W.Y., Kim D.W., Kim H.J., Kim K., Lee S.H., Bae J.H., Kang I.M., Kim K., Jang J. (2022). Environmentally and electrically stable sol-gel-deposited SnO_2_ thin-film transistors with controlled passivation layer diffusion penetration depth that minimizes mobility degradation. ACS Appl. Mater. Interfaces.

[20] Fortunato E., Barquinha P., Martins R. (2012). Oxide semiconductor thin-film transistors: A review of recent advances. Adv. Mater..

[21] Aharonyan K.H., Kazaryan E.M., Bazzan M., Kokanyan E.P. (2024). Impurity affected transport properties of quantum well heterostructures with electronic and high-κ dielectric quantum screening. Physica B.

[22] Bierwagen O., Speck J.S. (2010). High electron mobility In_2_O_3_ (001) and (111) thin films with nondegenerate electron concentration. Appl. Phys. Lett..

[23] Lee C., Lee W.Y., Kim D.W., Kim H.J., Bae J.H., Kang I.M., Lim D., Kim K., Jang J. (2021). Extremely bias stress stable enhancement mode sol-gel-processed SnO_2_ thin-film transistors with Y_2_O_3_ passivation layers. Appl. Surf. Sci..

[24] Lee C., Lee W.Y., Lee H., Ha S., Bae J.H., Kang I.M., Kang H., Kim K., Jang J. (2020). Sol-gel processed yttrium-doped SnO_2_ thin film transistors. Electronics.

[25] Cho Y., Lee S., Heo S., Choi S., Bae J.H., Kang I.M., Kim K., Lee W.Y., Jang J. (2025). Electrically and environmentally stable nitric acid-assisted SnO_2_ films for the active channel layer of thin-film transistors. Mater. Today Adv..

[26] Bang S., Lee S., Park J., Park S., Ko Y., Choi C., Chang H., Park H., Jeon H. (2011). The effects of post-annealing on the performance of ZnO thin film transistors. Thin Solid Films.

[27] Hesse R., Streubel P., Szargan R. (2007). Product or sum: Comparative tests of Voigt, and product or sum of Gaussian and Lorentzian functions in the fitting of synthetic Voigt-based X-ray photoelectron spectra. Surf. Interface Anal..

[28] Manceriu L.M., Maho A., Labrugere C., Tixhon E., Schrijnemakers A., Rougier A., Colson P., Cloots R. (2020). Influence of quenching on the opto-electronic properties of F:SnO_2_ layers. ACS Omega.

[29] Kirmani A.R., Roe E.F., Stafford C.M., Richter L.J. (2020). Role of the electronically-active amorphous state in low-temperature processed In_2_O_3_ thin-film transistors. Mater. Adv..

[30] Tiwale N., Subramanian A., Dai Z., Sikder S., Sadowski J.T., Nam C.-Y. (2020). Large mobility modulation in ultrathin amorphous titanium oxide transistors. Commun. Mater..

[31] Avis C., Jang J. (2022). Understanding the origin of the hysteresis of high-performance solution processed polycrystalline SnO_2_ thin-film transistors and applications to circuits. Membranes.

[32] Tauc J., Grigorovici R., Vancu A. (1966). Optical properties and electronic structure of amorphous germanium. Phys. Status Solidi B.

[33] Yusuf A.S., Ibrahim S.O., Mohammed I.K., Ahmadu U., Isah K.U. (2026). Effects of deposition parameters on optical and electrical properties of colloidal SnO_2_ film. Discov. Electron..

[34] Nomura K., Ohta H., Takagi A., Kamiya T., Hirano M., Hosono H. (2004). Room-temperature fabrication of transparent flexible thin-film transistors using amorphous oxide semiconductors. Nature.

[35] Baddorf A.P. (2023). Identifying the secondary electron cutoff in ultraviolet photoemission spectra for work function measurements of non-ideal surfaces. Sci. Rep..

[36] Helander M.G., Greiner M.T., Wang Z.B., Lu Z.H. (2010). Pitfalls in measuring work function using photoelectron spectroscopy. Appl. Surf. Sci..

[37] Hoel C.A., Mason T.O., Gaillard J.F., Poeppelmeier K.R. (2010). Transparent conducting oxides in the ZnO-In_2_O_3_-SnO_2_ system. Chem. Mater..

[38] Faber H., Das S., Lin Y.H., Pliatsikas N., Zhao K., Kehagias T., Dimitrakopulos G., Amassian A., Patsalas P.A., Anthopoulos T.D. (2017). Heterojunction oxide thin-film transistors with unprecedented electron mobility grown from solution. Sci. Adv..

[39] Wen P., Peng C., Ding X., Chen F.H., Yan G., Xu L., Li J., Li X., Zhang J. (2025). High mobility crystallized stacked-channel thin-film transistors induced by low-temperature thermal annealing. Appl. Phys. Lett..

[40] Lee J., Chung D.S. (2023). Heterojunction oxide thin film transistors: A review of recent advances. J. Mater. Chem. C.

[41] Han J.H., Shin D.Y., Sung C., Cho S.H., Ju B.K., Chung K.B., Nam S. (2024). Tuning the threshold voltage of an oxide thin-film transistor by electron injection control using a p-n semiconductor heterojunction structure. ACS Appl. Mater. Interfaces.

[42] Son K.S., Kim T.S., Jung J.S., Ryu M.K., Park K.B., Yoo B.W., Park K., Kwon J.Y., Lee S.Y., Kim J.M. (2009). Threshold voltage control of amorphous gallium indium zinc oxide TFTs by suppressing back-channel current. Electrochem. Solid-State Lett..

[43] Cheong W.S., Chung S.M., Shin J.H., Hwang C.S. (2011). Electrical properties of top-gate oxide thin-film transistors with double-channel layers. J. Cryst. Growth.

[44] Lee J., Eun J.S., Na J.H., Park W., Park J.H., Feng J., Jang J., Kang I.M., Park J., Zhang X. (2024). Verifying the physical role of upper-active-layer on charge transport together with bias stability in bilayer-channel oxide thin-film transistors. Surf. Interfaces.

